# The Impact of Loadshedding on the Psychosocial Well-Being of Older Adults in Residential Facilities in South Africa: Implications for Gerontological Social Services

**DOI:** 10.1007/s10823-026-09606-8

**Published:** 2026-11-09

**Authors:** Gabriella Wysoke, Stephan Geyer, Marien Alet Graham

**Affiliations:** 1 Department of Social Work and Criminology, University of Pretoria, Pretoria, South Africa; 2 Department of Mathematics Education, University of South Africa, Pretoria, South Africa

**Keywords:** Loadshedding, Loneliness, Older adults, Resilience, Stress, South Africa

## Abstract

Planned rolling electricity outages in South Africa when electricity demand exceeds supply (“loadshedding”) have a multifaceted impact on the population. To the authors’ knowledge, the impact of loadshedding on the psychosocial well-being of South African older adults (60+) in residential facilities has not been investigated. The phenomenon was considered from an ecosystem lens. The study aimed to determine the impact of loadshedding on the psychosocial well-being of South African older adults in residential care and provide guidelines for gerontological social services. Quantitative research utilising a cross-sectional design and non-probability sampling was undertaken. Face-to-face surveys were conducted with 68 older adults at five facilities, both public and private, in the Gauteng Province of South Africa. Stress (Perceived Stress Scale), resilience (Brief Resilience Scale), and loneliness (Brief Loneliness Scale) were investigated. Participants reported moderate levels of perceived stress (*M* = 17.7) and moderate feelings of loneliness (*M* = 14.7) during loadshedding. A significant proportion of participants (67.6%) reported that loadshedding affected their psychosocial well-being. Participants’ resilience scores were within the “normal” range (*M* = 3.7) regarding loadshedding. The experiences reported by older adults in residential facilities suggested associations with feelings of stress and loneliness during loadshedding. Recommendations for individual psychosocial support, group support among similarly affected older adults, advocacy, and long-term solutions to secure alternative power supplies that enable older adults to resile amid loadshedding are offered.

## Introduction

Globally, the population of older adults is growing, with estimates that by 2050, there will be 2.1 billion people worldwide aged 60 years and older (World Health Organization, 2022). Projections suggest that by 2030, the older population in South Africa, the country where the research reported on was undertaken, will reach 7 million, with current estimates indicating that there are 6.6 million older adults (aged 60 and above) in the population of 63.1 million in South Africa (Statistics South Africa, 2025). The growing population of older adults, both in South Africa and globally, highlights the need for human services that address their psychosocial well-being.

The psychosocial well-being of older adults in South Africa can be considered “a multidimensional construct consisting of psychological, social, and subjective components which influence the overall functionality of individuals in achieving their true potentials as members of the society” (Kumar, 2020, p. 676). Psychosocial well-being is influenced by factors such as loss of autonomy, functional disability, and the death of peers (Kopylova et al., 2022). Good health, functional abilities, and economic stability serve as mediators of greater happiness (Chirinda & Phaswana-Mafuya, 2019). Health factors, such as loss of hearing, may also be associated with a greater prevalence of loneliness, isolation, and depression (de Andrade et al., 2022). Approximately 76% of older adults in South Africa live below the poverty line and 3.1 million older adults’ income is limited to the South African Social Security Agency (SASSA) old age grant of R2 310 (roughly US$140.68, at the exchange rate of R16.42 to $1) per month (Kopylova et al., 2022; South African Government, 2025; South African Human Rights Commission, 2019; Wise.com, 2026). Just over 18,000 older adults in South Africa live in residential facilities. These are structures designed to provide accommodation for older adults, including independent housing schemes, residential lodging, assisted living, and frail care (The Association for the Aged, 2024). It should be noted that racial disparities in residential care may be ascribed to South Africa’s history of racial segregation (“Apartheid”), whereby only white older adults were able to access residential facilities until the end of Apartheid in 1994 and the passing of the Transformation of Certain Rural Areas Act 94 of 1998 (Hlongwane, 2024). Long-term care has not yet been fully transformed, mainly due to historical inequalities and a focus on community-based care within African communities (Hlongwane, 2024).

Ageing is often linked to various psychological and social challenges, such as loneliness, stress, and isolation (Chipps & Jarvis, 2016), which stressors like loadshedding may exacerbate. Loadshedding refers to planned rolling electricity outages in South Africa, implemented when electricity demand exceeds supply, and is considered a means of ensuring a controlled rotation of electricity supply (City of Johannesburg, 2018). South Africa also experiences intermittent electricity supply due to substation failures caused by poor maintenance or vandalism, as well as the theft of power cables, leaving consumers without electricity for days or weeks. In South Africa, loadshedding is implemented in stages, ranging from Stage 1 to Stage 8, depending on the severity of the power shortage, with higher stages indicating more extended periods without electricity (Western Cape Government, 2019). In 2023, South Africa experienced 6,947 hours of loadshedding, while in 2024, there were 1,656 hours of loadshedding (The Outlier, 2024).

The introduction of loadshedding since 2008 and other electricity-related difficulties in South Africa have had an undeniable impact on the population, including older adults. Older adults and people with disabilities are particularly vulnerable due to mobility impairments, chronic and comorbid illnesses, and reliance on equipment that uses electricity, such as oxygen machines (Marchetti-Mercer, 2023). While access to basic services, such as electricity, affects all South African older adults, the present study focused on those in residential facilities, bearing in mind the additional challenges they may face that exacerbate the impact of loadshedding. For example, older adults in residential care often cannot rely on the same power alternatives as their peers residing in the community; for example, for safety reasons, residential facilities prohibit the use of candles, gas/paraffin heating/ovens in rooms, or open fires. Although the study reports on South African older adults, loadshedding is implemented in countries such as Ghana, Zimbabwe, Zambia, and Pakistan (Malik et al., 2022; Matewe & Samson, 2021; Moore, 2019; Umar & Kunda-Wamuwi, 2019). Furthermore, natural disasters such as floods, tornadoes, hurricanes, and wildfires destroy infrastructure, leaving citizens without access to electricity. This can be seen in recent extreme weather events in the United States, where in 2024 and 2025 alone, hundreds of thousands of Los Angeles residents were left without electricity due to wildfires, and five million citizens were left without electricity due to Hurricane Helene (Scharping, 2025). It has been reported that the US power network is vulnerable to increasingly frequent extreme weather events, with similar warnings issued in the United Kingdom (Kuriakose & Wood, 2025), Europe (Beyond Fossil Fuels, 2025), and China (Reuters, 2025), among others. Thus, the study may be of interest to a broader audience where electricity interruptions occur.

Loadshedding has been linked to psychological challenges amongst South Africans of all ages, including stress, feelings of depression, anxiety, and frustration (Marchetti-Mercer et al., 2024; The South African Depression and Anxiety Group, 2023). Loadshedding may also lead to feelings of mental strain, isolation, a lack of safety, and anger (Marchetti-Mercer et al., 2024).

Socially, the implementation of loadshedding in South Africa has also been associated with domestic disruptions, increasing rates of particular crimes (such as assault, robbery, and sexual assault), financial strain due to rising prices of consumer goods, disruption of social unity, exacerbation of problems for marginalised groups (such as people living in poverty, women, and older adults), and disruptions to water and sanitation systems (Booysen et al., 2023; City of Johannesburg, 2020; Inglesi-Lotz, 2023; Marchetti-Mercer, 2023; Marchetti-Mercer et al., 2024; The South African Depression and Anxiety Group, 2023).

Despite the adverse effects of loadshedding, there remains little research on these effects among older adults. Strategies recommended by South African psychologists for coping with loadshedding include being optimistic, connecting with others, maintaining a routine, expressing feelings, engaging in enjoyable activities (Lourens, 2023), preparing for loadshedding, adhering to a regular sleep schedule, and using loadshedding time constructively (Lownie, 2019). Marchetti-Mercer et al. (2024) emphasised the importance of addressing the issue of loadshedding and its impact on individuals, taking into account the various socioeconomic factors that exacerbate the effects of loadshedding on specific groups. However, these recommendations are not specific to older adults, especially those in residential facilities.

It was confirmed, after searches of various databases and research platforms (e.g., EBSCOHost, Google Scholar, PsycINFO, and Sabinet African Journals), that there is a distinct lack of research establishing the specific impact of loadshedding on South African older adults’ psychosocial well-being, especially among those residing in residential facilities. While other studies have established the impact of loadshedding broadly or on other populations (Banderker, 2022; Bantjes & Swartz, 2023; Marchetti-Mercer et al., 2024), there remains a gap in understanding its impact on older adults as a specific population group. As such, the study reported on was conducted to determine the impact of loadshedding on South African older adults’ psychosocial well-being. In the study reported, psychosocial well-being was assessed using measures of stress, loneliness, and resilience, rather than more traditional indicators such as depression, social support, and quality of life.

Due to the limited literature on this topic, indicators of stress, resilience, and loneliness were used to better align with other South African literature on loadshedding. For example, Banderker (2022), Kgarose et al. (2023), Marchetti-Mercer et al. (2024), Mashiyane et al. (2024), Steenkamp et al. (2016) and The South African Depression and Anxiety Group (2023), among others, all identify and discuss South Africans’ experiences of stress during periods of loadshedding. Perceived stress, as included in the study, is closely related to uncertainty about and responses to an unpredictable, continuous stressor (Cohen et al., 1983), such as loadshedding.

Furthermore, it is important not to conflate psychological distress during periods of loadshedding with the severe and persistent disturbances in thinking and functioning of diagnosed mental health disorders, like depression (Bantjes & Swartz, 2023). Depression is a clinically diagnosed mental health disorder with multi-faceted causes that would be difficult to attribute to loadshedding alone. Marchetti-Mercer et al. (2024) also emphasise the difficulty in establishing causal links between loadshedding and mental health disorders.

It should also be noted that loneliness is a separate concept to social support, with social support often being a predictor of loneliness (Zhang & Dong, 2022). While loadshedding often disrupts events, as discussed in this article (e.g., meal times and venues in residential facilities, religious services, and routines), it usually does not alter the broader network of social support available to older adults outside periods of loadshedding. Experiences of loneliness, as a measure of feeling a loss of connection, can change over short periods (specifically during loadshedding) and thus may more accurately reflect the experiences of older adults during loadshedding, as opposed to a long-term, structural measure like social support. Loneliness as an indicator has also been noted among the older population during national-level disruptions, such as the COVID-19 pandemic (Geyer et al., 2023), which may relate more closely to experiences of loadshedding.

Quality of life is a broad, multi-dimensional construct that is shaped by many different factors (Kopylova et al., 2022), and thus, may not have fully reflected the impact on older adults relating to one specific environmental factor (i.e., loadshedding).

The inclusion of resilience speaks to South Africa’s broader policies for social welfare services, like the White Paper for Social Welfare (Department of Welfare, 1997) and the Older Persons Act 13 of 2006 (Republic of South Africa, 2006), which include the identifying of targets for social services rendered by human service professionals, and strengthening older adults’ resilience and capacity to cope with environmental stressors, like loadshedding. Hence, the inclusion of resilience allows human service professionals to assess older adults’ resilience in the face of loadshedding, identify those requiring additional support, and work with them to build their capacity to cope with loadshedding and its associated effects.

The study was underpinned by the ecosystem theory, which examines the individual’s environment in terms of various intersecting and embedded systems (Mbedzi, 2019). The ecosystem theory informed the interpretation of results and the recommendations provided, rather than the measurements alone. The intersecting and embedded systems include the microsystem, in which the individual plays a direct role. The systems also include the mesosystem, in which microsystems interact; the exosystem, which the individual is not directly included in but still influences their lives; the macrosystem, which encompasses the broader society or environment; and the chronosystem, which depicts changes over time (Louw & Louw, 2014).

The macrosystem condition of loadshedding would be expected to relate to older adults’ psychosocial well-being. For example, microsystem processes, such as mealtimes and medical assistance devices, could be disrupted by loadshedding, which may exacerbate feelings of stress among older adults. Disrupted or cancelled activities and a lack of opportunities for socialisation, which occur in the mesosystem, could also be affected by loadshedding, which may, in turn, shape older adults’ experiences of resilience and loneliness.

This allowed for an understanding of loadshedding’s impact (as a macrosystem policy) that extended beyond the individual, microsystem effects such as stress, to include the impact of loadshedding on mesosystem transactions, and exosystem interactions within the residential facilities. As such, the recommendations offered do not focus solely on social services in the microsystem (individual remediation) but also include interventions in the mesosystem (group work), exosystem (advocacy), and macrosystem (long-term solutions to loadshedding). These recommendations may be transferable to other settings where similar electricity interruptions occur.

## Materials and Methods

A quantitative study, operationalised through a cross-sectional survey design, was implemented to investigate the impact of loadshedding on the psychosocial well-being of older adults residing in residential facilities.

### Population and Sampling

The study population consisted of older adults residing in both public (i.e., government-owned) and private (i.e., non-profit organisations partially subsidised by the government) residential facilities across the three metropolitan municipalities in Gauteng, namely, Ekurhuleni, Johannesburg, and Tshwane. Gauteng is one of South Africa’s nine provinces and is home to the largest number of older adults in the country, i.e., 1.4 million (Statistics South Africa, 2023). The first author purposively selected five residential facilities, three public and two private. In Ekurhuleni, one private and one public residential facility were research sites, both of which provided varying levels of long-term care (independent living in rooms, assisted living, and frail care) based on individual needs. In Johannesburg, one public residential facility providing subsidised housing for older adults was included as a research site. In Tshwane, one private and one public residential facility were included as research sites, both of which provided varying levels of long-term care (independent living in rooms, assisted living, and frail care) based on the needs of individual residents. Of the three public residential facilities, two had diesel-powered generators used only for emergency lighting during loadshedding, while the remaining three had no backup power. After securing permission from each research site, the sampling procedure followed a two-phase, indirect, non-probability sampling approach. The first author contacted the managers or social workers at the residential facilities, who provided permission for the study to be conducted and were furnished with the study’s inclusion criteria, as well as an information sheet to be displayed on a notice board or in a communal area within the facility. The first author requested that the managers or social workers at each facility identify approximately 15 residents who were able and willing to participate in the study, yielding an estimated total of 75 participants across the residential facilities. Furthermore, the first author requested that the information sheet be displayed at the residential facilities so that interested residents could approach the manager or social worker to put their names forward as participants. Thus, participants had two options for declaring their willingness to participate in the study: either through purposive sampling or through volunteer sampling. The number of residents who were approached, screened, or declined to participate is unknown to the authors due to the Protection of Personal Information Act 4 of 2013, which prohibits direct recruitment. Hence, indirect purposive sampling (Maree & Pietersen 2020a) was utilised, in accordance with the Act. The first author conducted face-to-face surveys with 68 older adults who expressed interest, were available, and met the inclusion criteria (aged 60 years or older, able to converse in English, without diagnosed forms of cognitive decline, residing in a facility affected by loadshedding, and able to provide written informed consent). Before contacting the first author for data collection, the managers or social workers at the facilities reviewed resident records to confirm the absence of diagnosed forms of cognitive decline. It should be noted that only English-speaking older adults were eligible to participate (see inclusion criteria), which may have affected diversity and representativeness.

### Instrument

Apart from sociodemographic questions, the instrument included three standardised scales in measuring the impact of loadshedding on the psychosocial well-being of older adults in residential care. The survey included the Perceived Stress Scale (PSS-10), Brief Resilience Scale (BRS), the Brief Loneliness Scale (BLS), and the impact of loadshedding on mental and social well-being. The survey also included a literature-based, self-developed 15-item anxiety symptoms checklist that has been omitted from this paper to limit the focus to standardised scales.

The PSS-10 was developed by Cohen et al. (1983) to assess how stressful a participant deems their life, or life in general, to be. This referred to how stressful participants perceived loadshedding to be. Using commonly adopted categories, scores of 0–13 indicate low perceived stress, 14–26 indicate moderate perceived stress, and 27–40 indicate high perceived stress (Bizri et al., 2022; Daraje et al., 2026). The PSS-10 demonstrated good internal reliability (α = 0.846). Furthermore, the PSS-10 has shown concurrent validity with depression and anxiety (Ezzati et al., 2014). The PSS-10 instruction was adapted for the study by including loadshedding therein (i.e., please listen to each item and indicate how frequently you have experienced these feelings during periods of loadshedding).

The BRS was developed by Smith et al. (2008) to assess the ability to recover from psychological stress and measures resilience as the ability to “bounce back” from stress, mainly when it is continuous (Smith et al., 2008, p. 194), as loadshedding is an ongoing situation in South Africa. A score of 1.00–2.99 indicates low resilience, 3.00–4.30 indicates normal resilience, and 4.31–5.00 indicates high resilience (Smith et al., 2013). The BRS demonstrated good internal reliability (α = 0.815). The developers of the BRS also confirmed its internal reliability (α = 0.80 − 0.91), test-retest reliability and convergent and discriminative predictive validity (Smith et al., 2008). The BRS instruction was adapted for the reported study by including loadshedding (i.e., consider each statement and choose the one most applicable to you during periods of loadshedding).

The BLS was developed by De Jong Gierveld and Van Tilburg (2010) to measure social, emotional, and overall loneliness, in this case resulting from loadshedding. The BLS consists of 6 items, which were operationalised using a 5-point Likert scale (1–5) in the reported study. This differs from the traditional dichotomous or 3-point Likert scale used by the BLS, but was chosen to capture greater variability in responses. The higher the mean score, the greater the loneliness experienced, with a theoretical range of 6 to 30. Based on the principle that higher BLS mean scores indicate greater loneliness, the authors interpreted the scores as follows: 6.0–14.0 as low, 14.1–22.0 as moderate, and 22.1–30.0 as high. These bands for interpretation were author-defined and therefore have not been validated. Furthermore, the BLS score is a sum score ranging from 6 to 30, and it is this total to which the cut-offs apply. In the study, the scale demonstrated good internal reliability (α = 0.712), consistent with the developers’ coefficients (α = 0.81 − 0.85). The developers further confirmed the scale’s convergent validity (De Jong Gierveld & Van Tilburg, 2010). The BLS instruction was adapted to include loadshedding (i.e., please rate each statement on the scale provided as you experience it during loadshedding).

As the instructions for the abovementioned standardised scales were adapted to specifically refer to loadshedding, published validity evidence and cut-offs may not transfer directly. Thus, categories must be interpreted with some caution.

Furthermore, the instrument included dichotomous (yes/no) questions regarding the psychosocial impact of loadshedding (i.e., do you feel that loadshedding affects your psychosocial well-being? If yes, please elaborate on how), the social impact of loadshedding (i.e., do you feel that loadshedding affects you socially – please elaborate), stress (i.e., do you experience stress during loadshedding? If yes, please elaborate), and loneliness (i.e., do you experience loneliness during periods of loadshedding – please elaborate).

### Data Collection

The study utilised face-to-face surveys, whereby the first author met with participants at each research site and completed the informed consent form and study with them (Maree & Pietersen 2020b). Data were collected between November and December 2024, not during a period of active loadshedding (as such, responses were retrospective). Thereafter, the data were captured in Qualtrics^XM^. Surveys were conducted exclusively in English, with participants’ proficiency levels established by the managers/social workers at the facilities who facilitated the recruitment​. No data were collected regarding the specific number of hours of loadshedding experienced at each facility. However, broad figures are available that South Africa experienced 6,947 hours of loadshedding in 2023 and 1,656 hours of loadshedding in 2024 (The Outlier, 2024).

### Data Analysis

The data analysis comprised descriptive and inferential statistics using IBM SPSS Statistics (Version 30) (IBM Corp., 2025) for data analysis by the third author. The data were tested for normality using the Shapiro-Wilk test; however, not all p-values were greater than 0.05, indicating that the normality assumption was violated. Hence, a non-parametric test, namely the Mann-Whitney U test (hereafter referred to as the MW test), was employed. The MW test was used to assess differences in responses to the scales across groups (Emerson, 2023). Regarding the computation of effect sizes for the MW test, effect sizes were calculated as r = z/sqrt(N), where z is the standardised Mann-Whitney U test statistic, and N is the total sample size. Effect sizes were interpreted as |r| ≥ 0.10 small, |r| ≥ 0.30 medium, and |r| ≥ 0.50 large (Peres, 2026). Statistically significant differences were interpreted at the 5% level, as is often the case in the social sciences (Emerson, 2023). Given the exploratory nature of this study, corrections for multiple comparisons, such as the Bonferroni adjustment, were not applied. Although such procedures reduce the likelihood of Type I errors, they have been criticised for being overly conservative, particularly in studies with relatively small samples, as they may substantially increase the risk of Type II errors and obscure potentially meaningful relationships (Barnett et al., 2022; Perneger, 1998). In the present study, the analyses were intended to explore patterns of differences in psychosocial outcomes, namely stress, resilience, and loneliness, across a range of demographic and loadshedding-related characteristics among older adults residing in residential facilities. Applying a strict Bonferroni correction would have reduced the ability to detect potentially important associations in this under-researched area. Consistent with recommendations for exploratory research, all analyses are reported transparently with exact p-values, effect sizes, and cautious interpretation of statistical significance rather than relying solely on dichotomous significance testing.

An approximate sensitivity analysis was conducted in Python 3.12 using SciPy 1.17; its assumptions and findings are reported in the limitations section.

Furthermore, content analysis was utilised, with participants asked to respond to open-ended questions. The first author reviewed participants’ responses when asked to elaborate on or describe their experiences to become familiar with the data, identified patterns and themes among responses with similar ideas and grouped them into categories (Nieuwenhuis, 2020). The second author reviewed the coding and categories and gave feedback, which was used to refine the categories (Nieuwenhuis, 2020).

## Results

The sociodemographic characteristics of the participants were explored. Furthermore, the self-reported psychosocial impact of loadshedding was measured, as well as stress, resilience, loneliness, and self-reported social impact. It should be noted that, in terms of psychosocial well-being, there is both a single self-report item (yes/no) and psychosocial well-being indicators which encompass the standardised scales reported on (BRS, PSS-10, and BLS). Due to the nature of face-to-face surveys, there were no missing data in the study.

### Sociodemographics

The participants (*N* = 68) exhibited diverse sociodemographic characteristics (see Table 1).

**Table 1:** Sociodemographic profile

Variables	*M (SD* *)*	*n* (%)
Continuous variable		
Age	77.1 (7.4)	
Categorical variables		
Gender		
Male		17 (25.0)
Female		51 (75.0)
Home language		
Afrikaans		34 (50.0)
English		30 (44.1)
Other		4 (5.9)
Educational qualification		
Obtained Grade 12		28 (41.2)
Completed some high school training		20 (29.4)
Higher certificate		9 (13.2)
Bachelor’s degree/Honours degree/Professional degree		6 (8.8)
Completed primary school		3 (4.4)
National diploma		1 (1.5)
Postgraduate qualification		1 (1.5)
Employment status		
Retired/Pensioner/Receiving old age grant		65 (95.6)
Self-employed/entrepreneur		1 (1.5)
Unemployed		2 (2.9)
Relationship status		
Widowed		30 (44.1)
Single		16 (23.5)
Divorced		14 (20.6)
Married/partnered		5 (7.4)
Other		3 (4.4)
Type of residential facility		
Independent living in a room or unit within a residential facility		47 (69.1)
Independent living in a housing scheme/subsidised housing		15 (22.1)
Frail care		4 (5.9)
Assisted living		2 (2.9)
Number of roommates		
0		37 (54.4)
1		26 (38.2)
2		3 (4.4)
3		1 (1.5)
4		1 (1.5)

The participants comprised a diverse group, with varying levels of education, relationship statuses, and numbers of roommates. These factors may affect experiences of loadshedding, for example, widowhood and having no roommates may affect opportunities for socialisation and coping. The limited source of income (“retired/pensioner/receiving old age grant”) may inhibit participants’ ability to obtain battery backups and expensive power alternatives.

### Psychosocial Well-Being, Stress, and Resilience

When asked with a dichotomous yes/no question if participants felt that loadshedding impacted their psychosocial well-being (and if so, how), the majority of participants indicated that loadshedding affected their psychosocial well-being negatively (67.6%), citing (amongst others), the following reasons to an open question: inconvenience and frustration (*n* = 15), boredom (*n* = 13), fear of crime (*n* = 12), anger (*n* = 8), sadness/depression (*n* = 8), stress (*n* = 8), anxiety (*n* = 5), and financial concerns (*n* = 5).

Participants indicated moderate levels of perceived stress during periods of loadshedding according to the PSS-10 (*M* = 17.7, *SD* = 9.9, 95% CI [15.30, 20.11]), with scores ranging from 2 to 38. Please refer to Table 2 for the categorical distribution of the PSS-10. This was reiterated by participants who, when asked with a dichotomous yes/no question, whether they experienced stress during loadshedding, stated that they experienced stress during loadshedding (54.4%).

**Table 2:** Categorical distribution of PSS-10

Category	*n*	%
Low perceived stress (0–13)	25	36.8
Moderate perceived stress (14–26)	27	39.7
High perceived stress (27–40)	16	23.5
Total	68	100

Participants indicated “normal” resilience, per the BRS (*M* = 3.7, *SD* = 0.9, 95% CI [3.44, 3.88]) during periods of loadshedding, with scores ranging from 1.2 to 4.8. Please refer to Table 3 for the categorical distribution of the BRS.

**Table 3:** Categorical distribution of BRS

Category	*n*	%
Low resilience (1.00–2.99)	15	22.1
Normal resilience (3.00–4.30)	32	47.1
High resilience (4.31–5.00)	21	30.9
Total	68	100

### Loneliness and Social Impact

Participants reported moderate loneliness during loadshedding according to the BLS (*M* = 14.7, *SD* = 5.7, 95% CI [13.32, 16.09]), with scores ranging from 6 to 28. This was affirmed by nearly half of the participants expressing loneliness during loadshedding (47.1%) when asked through a dichotomous yes/no question if they experienced loneliness during loadshedding, and to elaborate on both yes/no responses. Protective factors against loneliness, ascertained through the responses to the aforementioned question, included the presence of a roommate, spouse, friends, or staff (*n* = 14), maintaining a busy schedule (*n* = 11), preferring solitude (*n* = 6), and a sense of community (*n* = 3).

When asked via a dichotomous yes/no question with elaboration requested for both responses, participants indicated feeling that their daily routines and participation in activities around the residential facilities were disrupted, citing inconveniences such as (amongst others), unsettling of routine (*n* = 35), leisure activities disrupted (*n* = 30), lack of hot water to wash (*n* = 28), cooking disrupted (*n* = 20), and mealtimes disrupted (*n* = 17).

### Comparison Between Groups

The MW test was used to test for differences between groups, namely (a) age groups, (b) gender, (c) presence of roommates, (d) public or private status of residential facility, (e) impact of loadshedding on psychosocial well-being, (f) social impact of loadshedding, and (g) loneliness on the responses to the BRS, PSS-10, and BLS. Only the impact of loadshedding on psychosocial well-being, socially affected participants, and loneliness during loadshedding showed statistically significant differences (i.e., *p* < .05).

The results indicated that there was a significant difference between the BRS scores of participants who self-identified that loadshedding had a negative impact on their psychosocial well-being (67.6%) (*Mdn* = 3.3; *IQR* = 1.5) and participants who did not feel that loadshedding had a negative impact on their psychosocial well-being (32.4%) (*Mdn* = 4.3; *IQR* = 0.7) (*p* < .001; *z* = − 4.3; *U* = 182.5; *r* = .52). This difference can be considered large (Peres, 2026). The median difference was − 1.0, while the Hodges–Lehmann (HL) estimate^1^ of the location difference was − 0.83 (95% CI [− 1.33, − 0.50]). Participants who felt that loadshedding had a negative impact on their psychosocial well-being had statistically significantly higher scores on perceived stress (*Mdn =* 20.5; *IQR =* 14.0) than their unaffected counterparts (*Mdn =* 9.0; *IQR =* 11.5) (*p <* .001; *z = −* 4.4; *U =* 174.0; *r* = .53). This difference can be interpreted as large (Peres, 2026). The median difference was 11.5, while the HL estimate of the location difference was 11.00 (95% CI [7.00, 16.00]).

There was a statistically significant difference between the BRS scores of participants who stated that loadshedding affected their social well-being (36.8%) (*Mdn* = 3.0; *IQR* = 1.8) and their socially unaffected counterparts (63.2%) (*Mdn* = 4.2; *IQR* = 0.8) (*p* < .001; *z* = − 3.9; *U* = 230.5; *r* = .48). This difference can be considered medium (Peres, 2026). The median difference was − 1.2, while the HL estimate of the location difference was − 1.00 (95% CI [− 1.50, − 0.50]). A further significant difference was identified between the levels of perceived stress of participants who felt socially impacted by loadshedding (*Mdn* = 28.0; *IQR* = 9.0) and their socially unaffected counterparts (*Mdn* = 14.0; *IQR* = 12.0) (*p* < .001; *z* = − 4.8; *U* = 158; *r* = .59). This difference can be considered large (Peres, 2026). The median difference was 14.0, while the HL estimate of the location difference was 13.00 (95% CI [9.00, 18.00]). Participants who felt socially affected by loadshedding had significantly higher median scores on the BLS (*Mdn* = 17.0; *IQR* = 9.5) than the participants who did not feel socially affected by loadshedding (*Mdn* = 12.0; *IQR* = 7.0) (*p* = .002; *z* = − 3.2; *U* = 289.5; *r* = .38). This can be interpreted as a medium difference (Peres, 2026). The median difference was 5.0, which was identical to the HL estimate of 5.00 (95% CI [2.00, 8.00]).

The results indicated that participants who reported feeling lonely during loadshedding (47.1%) had significantly lower BRS scores (*Mdn* = 3.3; *IQR* = 1.5) than those who did not report feeling lonely (52.9%) (*Mdn* = 4.2; *IQR* = 1.9) (*p* = .001; *z* = − 3.5; *U* = 294; *r* = .42). This can be considered a medium difference (Peres, 2026). The median difference was − 0.9, while the HL estimate of the location difference was − 0.67 (95% CI [− 1.17, − 0.33]). There was also a significant difference in the PSS-10 scores of participants who felt lonely during loadshedding (*Mdn* = 25.5; *IQR* = 10.8) and participants who did not feel lonely during loadshedding (*Mdn* = 10.0; *IQR* = 11.3) (*p* < .001; *z* = − 5.2; *U* = 150.5; *r* = .64). This difference can be interpreted as large (Peres, 2026). The median difference was 15.5, while the HL estimate of the location difference was 13.00 (95% CI [9.00, 17.00]). Furthermore, the median score for the BLS among participants who felt lonely during loadshedding (*Mdn* = 17.5; *IQR* = 8.0) differed significantly from that obtained by participants who did not feel lonely during loadshedding (*Mdn* = 11.0; *IQR* = 6.0) (*p* < .001; *z* = − 5.2; *U* = 150.5; *r* = .64). This difference can also be interpreted as large (Peres, 2026). The median difference was 6.5, while the HL estimate of the location difference was 7.00 (95% CI [5.00, 9.00]).

## Discussion

The predominantly female sample reflects broader South African trends (Statistics South Africa, 2023). However, it does limit the data that could be obtained from males, which should be borne in mind when interpreting the results. The home languages indicated by respondents are not necessarily representative of those in Gauteng, where the most widely spoken languages are isiZulu, Sesotho, and Sepedi (Khanyile, 2022). The language disparities indicated may be ascribed to South Africa’s history of racial segregation (“Apartheid”) and lack of transformation of long-term care (Hlongwane, 2024).

To the authors’ knowledge, this study is the first to explore and describe the impact of loadshedding on older adults specifically; therefore, there is limited literature available for comparison. As such, comparisons are made using South African literature across all age groups, as well as literature from other countries.

The general negative psychosocial experience of participants in response to loadshedding due to inconvenience and frustration, boredom, fear of crime, anger, sadness/depression, stress, and financial concerns, and the experiencing of moderate perceived stress amongst participants reflect broader trends in South Africa. The South African Depression and Anxiety Group (SADAG) reported that, of the 1,836 participants of their survey, 42% felt depressed, and 73% feared crime during loadshedding (The South African Depression and Anxiety Group, 2023). Marchetti-Mercer et al. (2024) emphasised that loadshedding is linked to stress, mental strain, isolation, and fear for personal safety. Prolonged exposure of South Africans to a lack of basic services, like electricity, has the potential to trigger the development of mental health conditions such as depression, anxiety, and post-traumatic stress disorder (Laher et al., 2019). This has been further examined in South Africa across different population groups (Banderker, 2022; Kgarose et al., 2023; Mashiyane et al., 2024; Steenkamp et al., 2016).

Participants also reported fear of crime during loadshedding. There have been reported increases in interpersonal crime during loadshedding (Lambongang, 2023; Ram, 2024), which has promoted fear among many South Africans, who avoid going out or engaging in any activities outside the house at night during loadshedding (Marchetti-Mercer et al., 2024).

Some individuals raised financial concerns about why they believed loadshedding negatively affected their psychosocial well-being. The financial strain created by loadshedding, particularly on vulnerable groups like older adults, has been noted (Inglesi-Lotz, 2023; Masinga & Madzivhandila, 2023), emphasising that lower-income households often find themselves unable to afford power backups (e.g., uninterruptible power supplies, solar panels, inverters and battery systems) and have pre-existing vulnerabilities worsened by loadshedding.

It is thus evident from the data that moderate perceived stress and negative impacts on psychosocial well-being are experienced across population groups in South Africa.

Resilience amongst participants during periods of loadshedding was in the “normal” range. A Belgian study of the impact of COVID-19 on the well-being and cognitive functioning of 640 older adults found that they displayed normal resilience (*M* = 3.3, *SD* = 0.7) in response to COVID-19 (De Pue et al., 2021). Belgian older adults’ resilience in responding to COVID-19 is thus similar to that of South African older adults in responding to loadshedding. This similarity may be due to stressors perceived as beyond individuals’ control, as well as to older adults’ life experience, wisdom, and the accumulation of coping mechanisms over their lives (MacLeod et al., 2016). This highlights the importance of recognising resilience as a foundation for addressing environmental stressors, such as loadshedding.

Moderate loneliness, as reported by participants, resembles feelings of isolation and reduced opportunities for socialisation during loadshedding experienced by other population groups in South Africa. Loadshedding is linked to isolation and reduced social interaction, among other difficulties (Banderker, 2022; Marchetti-Mercer et al., 2024). The results of the BLS are similar to a quantitative cross-sectional study in South Africa that utilised the De Jong Gierveld 11-item loneliness scale to measure social, emotional, and overall loneliness among 118 older adults before and during COVID-19 (Geyer et al., 2023). During the COVID-19 pandemic, the mean score for overall loneliness was 32.3 (*M* = 32.3, *SD* = 10.8) among the participants, indicating moderate levels of loneliness (Geyer et al., 2023). While the mean scores between the two studies cannot be compared directly, the interpretation and categorisation of the mean scores as moderate levels of loneliness are compared. As such, the loneliness response to COVID-19 is reflected in the trend among participants in the present study who reported moderate loneliness in response to loadshedding.

Protective factors against loneliness included the presence of a spouse, roommate, friend, or staff member. This reiterates the findings of a study of the socioeconomic effects of loadshedding on poor urban households and small businesses in Zambia, wherein it was found that marital relations improved due to more time being spent together (Umar & Kunda-Wamuwi, 2019).

Participants in the study who kept themselves busy during loadshedding to avoid loneliness echo the participants in the SADAG survey, who reported planning their time effectively around loadshedding (Dworzanowski-Venter, 2023; The South African Depression and Anxiety Group, 2023). These participants were identified as being more resilient and experiencing less worry than those who used other, reactive coping skills (Dworzanowski-Venter, 2023; The South African Depression and Anxiety Group, 2023). This provides a parallel between the study’s results and those of other South Africans.

Participants also highlighted acceptance and community as protective factors against loneliness. In their analysis of humour used by South Africans to cope with loadshedding, Hurst and Donian (2024) explained that the humour used conveys a communal sense of being neglected, overlooked, and angry, as well as trauma bonding (Hurst & Donian, 2024). This is reflected in the participants who felt they had accepted the reality of loadshedding and those who felt a sense of community with other residents in the facility.

These results suggest a perceived negative impact during loadshedding on the psychosocial well-being of older adults and highlight potential avenues for various human service professionals, such as social workers, psychologists, and occupational therapists, to explore in addressing loneliness and isolation. These include establishing support groups and encouraging positive and proactive coping skills, such as acceptance and self-distraction.

Participants’ feelings that their daily routines and participation in activities around the residential facilities were disrupted due to the unsettling of routine, leisure activities being disrupted, a lack of hot water for washing, cooking being disrupted, mealtimes being disrupted, and sleep schedules being disturbed echo sentiments expressed by other groups affected by loadshedding.

The disruption of leisure activities due to loadshedding is reflected in South African and international studies. Butgereit (2015) reported that many South Africans expressed anger towards Eskom (the national, and only electricity supplier in the country) as loadshedding interrupted leisure activities, such as watching television. Similar findings are echoed in Zambia (Umar et al., 2021; Umar & Kunda-Wamuwi, 2019).

The SADAG found that 10% of participants were experiencing domestic routine disruptions (The South African Depression and Anxiety Group, 2023). Marchetti-Mercer et al. (2024) corroborated this, noting that loadshedding affects people’s ability to complete activities of daily living, work, study, and maintain relationships. Anger was also caused by difficulties with cooking and bathing, and by the inability to charge electronic devices (Butgereit, 2015). The experiences of participants thus reflect trends in South Africa regarding the routine disruptions caused by loadshedding.

Participants also alluded to the effects of loadshedding on access to water and sanitation. In South Africa, continuous loadshedding affects the water supply, as electricity is needed to pump water and water suppliers are not exempt from loadshedding (City of Johannesburg, 2020). The participants’ primary concern regarding water, however, was not a lack of supply but rather that loadshedding affects the geysers’ operation in the facilities, leading to a lack of hot water.

Participants felt that loadshedding disrupted their cooking and household tasks. Difficulties cooking or preparing food during loadshedding were identified by women in Zambia (Umar & Kunda-Wamuwi, 2019). Participants in a study examining the impact of loadshedding on people’s daily routines and functioning in Pakistan reported difficulties with cooking, procrastination, and incomplete tasks, as well as challenges with washing (Lodhi & Malik, 2013). There is therefore consensus between participants and the feelings of people in other countries affected by loadshedding.

The effects of loadshedding on older adults’ psychosocial well-being, as expressed by participants, highlight that loadshedding’s impact is pervasive and occurs across multiple systems. As such, interventions addressing loadshedding cannot only address the interpersonal effects of loadshedding but must focus on long-term, broader solutions to loadshedding itself.

The results indicated that there was a significant difference between the BRS and PSS-10 scores of participants who self-identified that loadshedding had a negative impact on their psychosocial well-being and participants who did not feel that loadshedding had a negative impact on their psychosocial well-being. A study of 426 Filipino adults during the COVID-19 pandemic found that higher perceived stress was associated with lower overall mental well-being (Acoba, 2024). In a study measuring resilience, the Big Five personality traits, positive and negative affect, perceived stress, and life satisfaction were examined in 288 Australian adults. The results revealed a relationship between the BRS and psychological well-being (McKay et al., 2021). These studies thus reflect the trend in the present study.

A statistically significant difference was identified between the BRS and PSS-10 scores of participants who felt that loadshedding affected them socially and their socially unaffected counterparts. Smith et al. (2008) found that the BRS was positively related to social relations. A cross-sectional study of 426 Filipino adults concluded that social support and perceived stress were negatively correlated (Acoba, 2024). Furthermore, the 6-item BLS, when used with 939 people in Brazil, was found to correlate negatively with social support (Coelho et al., 2018). These findings are thus consistent with the results of the present study.

There was also a significant difference in the BRS, PSS-10, and BLS scores of participants who reported feeling lonely during loadshedding and those who reported not feeling lonely during loadshedding. A cross-sectional study of 1,028 university students in Türkiye investigated the relationship between loneliness, perceived social support, and psychological resilience, finding a negative correlation between loneliness and the BRS (Öztürk & Kundakçı, 2021). A cross-sectional study of the associations of perceived stress with loneliness and depressive symptoms among 734 people in China concluded that higher perceived stress was associated with greater loneliness (Wang et al., 2024). These trends are reflected in the present study.

The statistically significant differences between groups highlight that older adults who experience greater stress, loneliness, and lower resilience can self-identify these negative experiences. As such, older adults should be encouraged to share their experiences of loadshedding and the difficulties they face, and to participate in the design and implementation of interventions addressing loadshedding across the micro, meso, exo, and macrosystems.

The study being reported on has filled a gap in the literature regarding the impact of loadshedding on the psychosocial well-being of older adults in residential facilities in South Africa. Furthermore, the study has provided recommendations for human service professionals to address the negative psychosocial impact of loadshedding amongst older adults. The experiences of older adults in other countries may differ. For example, the scheduled and staged nature of loadshedding, fear of crime, and legacy of Apartheid and racial segregation in residential facilities may be specific to South Africa, while the stress, loneliness, disruption of routine, and reliance on facility-level resources like geysers and alternative electricity supply may be relevant in other countries. Nevertheless, the experiences and recommendations herein may be relevant to older adults and human service professionals there, bearing in mind increasingly common extreme weather events and energy insecurity.

## Limitations

The quantitative research approach and empirical data collected may omit more detailed and personal information regarding participants’ experiences of loadshedding. The relatively small sample size (*N* = 68) is an important limitation of the study. The sample size limits statistical power and increases the possibility that smaller group differences or associations were not detected. An approximate sensitivity analysis for the Mann–Whitney U test was conducted using Python 3.12 with SciPy 1.17, assuming an equal-variance normal location-shift model. Based on the observed group sizes across the seven comparisons, the minimum detectable effects at 80% power and α = 0.05 corresponded to approximately *r* = .33 on the *r* = z/√N scale used in this study. Smaller effects may therefore have gone undetected, and non-significant findings should be interpreted cautiously rather than as evidence that no differences exist. The sample was not randomly selected and is therefore not representative, yet the study was exploratory in nature. Anxiety as a measure of psychosocial well-being was excluded from this article as the study did not include a standardised instrument to measure anxiety. The impact on residential facilities in urban areas in Gauteng Province, specifically, may differ from the impact of loadshedding in rural areas due to infrastructural and service delivery challenges. The data were collected at a single point in time; hence, the study does not report on possible long-term impacts of loadshedding. Nonetheless, it offers baseline data to inform gerontological social services. There was also no facility-level data on loadshedding exposure. It should be noted that older adults who felt more affected by loadshedding may have been more likely to volunteer to participate in the study, which may have affected the results. Further, only English-speaking older adults were included in the study, as the survey was only offered in English, which limited the diversity, representativeness, and generalisability of the sample and results. The indirect recruitment of participants may also have introduced bias into the selection process. The scales and measures utilised in the study relied on self-report and were retrospective, and may therefore have been affected by recall bias, response bias, or social desirability bias. Of the five facilities involved, two had diesel-powered generators that supplied emergency lights during loadshedding, which may have affected how loadshedding was felt by residents at those facilities.

## Conclusion: Implications for Gerontological Social Services

The study affirmed that South African older adults in residential facilities may self-report negative effects on their psychosocial well-being during loadshedding, including stress, loneliness, and the disruption of daily routines. Despite these challenges, participants indicated “normal” levels of resilience. It is evident that older adults who are particularly affected can self-identify their negative experiences and should thus be included in the design and implementation of interventions addressing loadshedding.

As such, it is recommended in the microsystem that human service professionals in residential facilities provide individualised psychosocial support to residents who are particularly impacted and who would benefit from such interventions. This includes interventions addressing the negative psychosocial impact (as expressed by 67.6% of participants) of loadshedding (such as the stress experienced by the majority of respondents and the PSS-10 mean score of 17.7, indicating moderate perceived stress) and the social impact of loadshedding (such as isolation and loneliness as reported above). Based on the BRS mean score indicating “normal” resilience during loadshedding, human service professionals should recognise the strength among older adults as a foundation for coping with loadshedding.

In the mesosystem, it is recommended that human service professionals establish support groups with residents who share similar experiences of psychosocial distress during loadshedding to create a support network that can be utilised as a source of strength and coping. Human service professionals should consider organising workshops or activities with residents during loadshedding hours, such as skills-based workshops, coping mechanisms workshops, or guest speaker sessions. This may assist in addressing the loneliness reported by 47.1% of participants and the BLS mean score of 14.7, which indicated moderate loneliness. Furthermore, these strategies may build on identified protective factors against loneliness, like the presence of roommates, spouses, friends, and staff.

Exosystemically, it is recommended that human service professionals, alongside older adults, take social action, empower older adults to mobilise, express the difficulties they face due to loadshedding, such as disrupted routines and activities, and advocate for systemic changes in South Africa to bring an end to loadshedding in the future.

At the macro level, private companies should be encouraged through tax incentives to help residential facilities obtain and maintain alternative electricity supplies, such as generators, solar panels, batteries, and inverter systems. This may assist in addressing macrosystem effects, such as a lack of hot water, financial concerns among participants, and disparities in access to alternative electricity supplies. Human service professionals should also receive training on identifying and monitoring loadshedding as a rights violation, as well as on the structures available in South Africa for reporting rights violations, such as the South African Human Rights Commission.

Furthermore, it is recommended that future research explore and describe the impact of loadshedding on the psychosocial well-being of community-dwelling older adults, which may then be utilised for comparative purposes. It is also recommended that future research utilise qualitative or mixed methods designs to allow for greater depth and the inclusion of older adults’ personal experiences. A longitudinal study may also be valuable for tracking potential changes in the effects of loadshedding on the psychosocial well-being of older adults over time.

## Data Availability

All data supporting the findings of this study are available within the paper.

## Footnotes

1 Note: The HL estimate represents the estimated location difference between the two groups based on the distribution of pairwise differences between observations. It therefore does not necessarily equal the simple difference between the two reported group medians. For example, where the reported group medians are 3.3 and 4.3, their difference is − 1.0, whereas the Hodges–Lehmann estimate may be − 0.83. The Hodges–Lehmann estimate is reported because it provides an appropriate estimate of the magnitude of the group difference for the non-parametric Mann–Whitney U test.
